# Parabanic acid is the singlet oxygen specific oxidation product of uric acid

**DOI:** 10.3164/jcbn.17-24

**Published:** 2017-09-05

**Authors:** Sayaka Iida, Yuki Ohkubo, Yorihiro Yamamoto, Akio Fujisawa

**Affiliations:** 1 School of Bioscience and Biotechnology, Tokyo University of Technology, 1404-1 Katakura-cho, Hachioji, Tokyo 192-0982, Japan

**Keywords:** singlet oxygen, uric acid, parabanic acid, oxaluric acid, sunlight exposure

## Abstract

Uric acid quenches singlet oxygen physically or reacts with it, but the oxidation product has not been previously characterized. The present study determined that the product is parabanic acid, which was confirmed by LC/TOFMS analysis. Parabanic acid was stable at acidic pH (<5.0), but hydrolyzed to oxaluric acid at neutral or alkaline pH. The total yields of parabanic acid and oxaluric acid based on consumed uric acid were ~100% in clean singlet oxygen production systems such as UVA irradiation of Rose Bengal and thermal decomposition of 3-(1,4-dihydro-1,4-epidioxy-4-methyl-1-naphthyl)propionic acid. However, the ratio of the amount of uric acid consumed to the total amount of singlet oxygen generated was less than 1/180, indicating that most of the singlet oxygen was physically quenched. The total yields of parabanic acid and oxaluric acid were high in the uric acid oxidation systems with hydrogen peroxide plus hypochlorite or peroxynitrite. They became less than a few percent in peroxyl radical-, hypochlorite- or peroxynitrite-induced oxidation of uric acid. These results suggest that parabanic acid could be an *in vivo* probe of singlet oxygen formation because of the wide distribution of uric acid in human tissues and extracellular spaces. In fact, sunlight exposure significantly increased human skin levels of parabanic acid.

## Introduction

Oxidative stress induces lipid peroxidation,^(1)^ DNA damage,^(2)^ and protein carbonylation,^(3)^ which can lead to diseases such as cancer,^(4)^ diabetes,^(5)^ Alzheimer’s disease,^(6)^ and ischemia reperfusion injury.^(7,8)^ Since initial oxidative stress is caused by various reactive oxygen species (ROS), the importance of identifying ROS *in vivo* is of interest in clinical investigations.

**Fig. 1:**
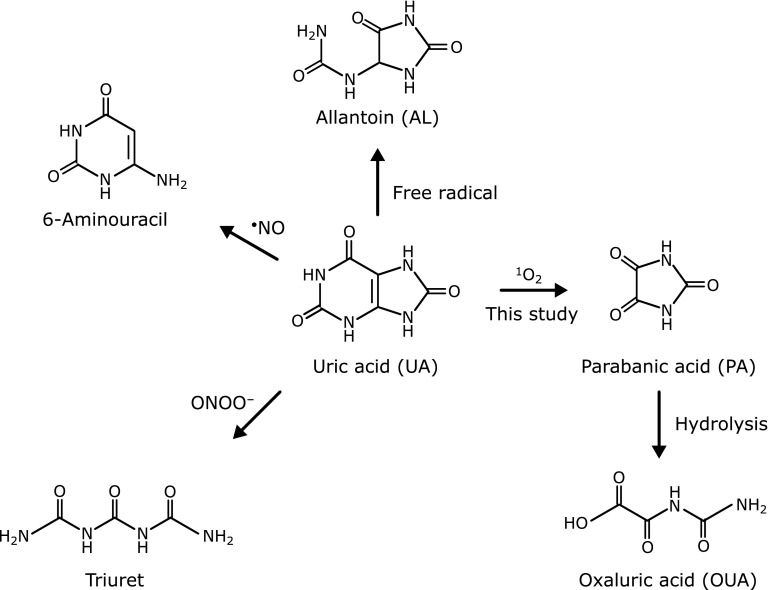
Reported oxidation products of UA induced by reactive oxygen species: AL is produced by free radical-induced oxidation; triuret by ONOO^−^; 6-aminouracil by ^•^NO; and PA by ^1^O_2_ (this study).

Identifying ROS *in vivo* can be done by monitoring an oxidation product as a marker. The oxidized substrate must show high reactivity toward different ROS and yield a specific oxidation product from an individual ROS. Uric acid (UA, Fig. 1) is an adequate substrate for this purpose. Uric acid is a terminal metabolite of purine in primates including humans. It is also a water-soluble antioxidant that can scavenge many types of ROS: free radicals,^(9)^ peroxynitrite (ONOO^−^),^(10)^ hypochlorous anion (ClO^−^),^(11)^ and singlet oxygen (^1^O_2_).^(9)^ Furthermore, its oxidation products are specific to the ROS (Fig. 1): free radical-induced oxidation gives allantoin (AL);^(12)^ ONOO^−^-induced oxidation yields triuret;^(13)^ and nitric oxide (^•^NO) gives 6-aminouracil.^(14)^ However, the ^1^O_2_ induced-oxidation product has not been identified.

Singlet oxygen (^1^O_2_) is a prominent ROS that plays an important role in bactericidal action. Nakano *et al.*^(15)^ showed that ^1^O_2_ killed *E. coli* effectively, although it was not harmful against human umbilical vein endothelial cells. Because the respiratory chains of eukaryotic cells are enclosed in mitochondria, whereas those of prokaryotic cells are contained in the cell membrane, ^1^O_2_ penetrating from the cell surface turns into harmless triplet molecular oxygen (^3^O_2_) before it reaches the mitochondria. Therefore, ^1^O_2_ can be considered a relatively innocuous ROS against eukaryotic cells. However, an excess amount of ^1^O_2_ can damage organisms, and some reports indicate that it causes oxidative damage to lipids,^(16)^ proteins,^(17)^ and DNA,^(18)^ and also induces apoptosis.^(19)^ Photosensitization is usually used to produce ^1^O_2_, but two-electron oxidation of H_2_O_2_ also can generate ^1^O_2_.^(20)^ The oxidation of H_2_O_2_ mimics myeloperoxidase (MPO), which produces ClO^−^ from H_2_O_2_ and Cl^−^. The ClO^−^ anion is a strong oxidant that can oxidize H_2_O_2_ to ^1^O_2_, ^(21)^ which suggests that ^1^O_2_ production may occur *in vivo* without sunlight exposure.

The current study demonstrated that parabanic acid (PA, Fig. 1) was formed specifically by ^1^O_2_-induced UA oxidation. Production of ^1^O_2_ resulted from thermal decomposition of 3-(1,4-dihydro-1,4-epidioxy-4-methyl-1-naphthyl)propionic acid (NEPO), photooxidation using Rose Bengal, and H_2_O_2_ oxidation by ClO^−^ or ONOO^−^, and PA was produced in high yield. However, the yield of PA was less than a few percent from peroxyl radical-, ClO^−^- or ONOO^−^-induced oxidation of UA. These results strongly suggest that PA is an oxidation product specific to ^1^O_2_ oxidation, and that PA and its hydrolysis product, oxaluric acid (OUA, Fig. 1), are suitable indicators of ^1^O_2_ production *in vivo*.

## Materials and Methods

### Chemicals

UA, PA, and other chemicals were purchased from Wako Pure Chemical Industries, Co., Ltd. (Osaka, Japan), Tokyo Chemical Industry Co., Ltd. (Tokyo, Japan), or Waken B Tech Co, Ltd. (Kyoto, Japan), and used as received. An ONOO^−^ generator, 3-(4-morpholinyl)sydnonimine hydrochloride (SIN-1), was purchased from Dojindo (Kumamoto, Japan). Authentic standard solutions of UA and PA were dissolved in 100 mM phosphate buffer (pH 7.4) and methanol, respectively, and stored at 4°C until use. The OUA was prepared by hydrolysis of PA upon addition of aqueous NH_3_ and then the solution was neutralized using 1 M HCl. The OUA formation was confirmed by LC/time-of-flight mass spectrometry (TOFMS) analysis using an ion corresponding to OUA (*m/z* = −131) and its fragment ion (*m/z* = −59).

ONOO^−^ was synthesized using a modified procedure described by Kato *et al.*^(22)^ Briefly, an ice-cold 0.7 M H_2_O_2_ solution containing 0.6 M HCl (10 ml) was added to a well-stirred 0.6 M NaNO_2_ solution (10 ml) in an ice bath, immediately followed by addition of 1.5 M NaOH (20 ml). The excess H_2_O_2_ was removed by addition of MnO_2_. The solution was then frozen at −25°C. The ONOO^−^ formed a yellow top layer due to frozen fractionation. This layer was collected and its concentration was determined as 330 mM by measuring its UV absorbance at 302 nm (ε = 1,670 M^−1^·cm^−1^).

### Oxidation of UA with ^1^O_2_ produced from the photo-irradiation of Rose Bengal

An aqueous mixture containing 50, 100, 150 or 200 µM UA and 10 µM Rose Bengal was irradiated by UVA light (1.12 mW/cm^2^) for ~3 h until all the UA was consumed. Next, the UVA light was turned off and the reaction solution was left at room temperature for ~9 h. Concentrations of UA and products were analyzed by LC/TOFMS and HPLC as described below.

### Oxidation of UA with ^1^O_2_ produced from NEPO

Thermal decomposition of NEPO produces ^1^O_2_. The purity of the NEPO was determined as 78% by the comparison of the UV absorption at 288 nm before and after the thermal decomposition of a methanolic solution of NEPO. Most of the NEPO was decomposed within 3 h at 35°C. A mixture of 50 or 100 µM UA and 8.0 mM NEPO in 50% aqueous methanol was incubated at 35°C for 3 or 12 h. Concentrations of UA and products were analyzed by LC/MS/MS and HPLC as described below.

### Oxidation of UA with ^1^O_2_ produced from H_2_O_2_ and NaClO or ONOO^−^

UA (130 µM) was dissolved with 2.5 mM H_2_O_2_ in 100 mM phosphate buffer (pH 7.4) containing 100 µM diethylenetriamine-*N*,*N*,*N'*,*N''*,*N''*-pentaacetic acid (DTPA) for chelation of transition metal ions. Next, 33 µl of 182 mM aqueous NaClO solution was added into 20 ml of the well-stirred reaction mixture at a constant rate (2 µM NaClO/min) using a syringe pump (Harvard Apparatus, Holliston, MA) over 2.5 h.

A methanolic SIN-1 solution (100 mM) or an aqueous ONOO^−^ solution (330 mM) was added to 100 mM phosphate buffer (pH 7.4) containing 150 µM UA, 2.5 mM H_2_O_2_, and 100 µM DTPA and incubated at room temperature for 3 h.

In the absence of H_2_O_2_, the oxidation of UA by NaClO or ONOO^−^ was carried out at room temperature for 3 h. Concentrations of UA and products were determined by HPLC as described below.

### UA oxidation by peroxyl radicals from 2',2-azobis(2-amidinopropane) dihydrochloride (AAPH)

A mixture of 150 µM UA and 10 mM AAPH in phosphate buffer solution (pH 7.4) containing 100 µM DTPA was incubated at 37°C for 3 h. Concentrations of UA and products were determined by HPLC as described below.

### Hydrolysis of PA to OUA

Phosphate buffers at pH 4.0 to 8.5 were prepared by adding 1 M NaOH or 1 M H_3_PO_4_ to the buffer solutions. Each 500 µM PA solution at various pHs was incubated under aerobic conditions at room temperature for 6 h. Concentrations of UA and OUA were determined by HPLC as described below.

### PA detection on human skin surface

Five healthy volunteers participated in this study after giving informed consent. Skin surface UA and OUA were collected from their forearms before and after exposure to sunlight for 2 h. The collection procedure was as follows. Five glass tubes containing 1.0 ml of methanol were prepared. The open end of each tube (ø 20 mm) was pressed tightly against the skin at different locations on the forearm and then rotated carefully to allow the methanol to contact the skin for 1 min. The extracts were combined and the solvent removed using a nitrogen gas flow. The residue was re-dissolved into methanol and analyzed using LC/MS/MS.

### HPLC analysis

The amounts of UA and its oxidative metabolites, PA, OUA, and AL, were determined by monitoring the absorption at 210 nm using a reversed-phase HPLC. The mobile phase was aqueous ammonium acetate (40 mM) and delivered at a rate of 1.0 ml/min. An ODS column (Tosoh, Tokyo, Japan; 5 µm, 4.6 mm × 250 mm) or a Develosil C30-UG (Nomura Chemical Co., Ltd., Tokyo, Japan; 5 µm, 250 mm × 4.6 mm) was used for separation. Retention times for UA, PA, OUA and AL were 7.8, 3.2, 11.0 and 2.5 min, respectively, using the ODS column, and 14.0, 7.0, 11.5 and 4.1 min, respectively, using the C-30 column.

### LC/TOFMS analysis

To obtain accurate mass-to-charge ratios (*m/z*) of UA oxidative metabolites, HPLC combined with TOFMS (JMS-T100LC, JEOL Ltd., Tokyo, Japan) was used. Negative ionization was performed at an ionization potential of −2,000 V. The optimized applied voltages to the ring lens, outer orifice, inner orifice, and ion guide were −5 V, −10 V, −5 V and −500 V respectively. To obtain accurate *m/z* values, trifluoroacetic acid (TFA) was used as an internal standard for *m/z* calibration.

### LC/MS/MS analysis

An LC/MS/MS system (LCMS-8040, Shimadzu, Kyoto, Japan) was used to determine the amounts of PA and OUA at the picomole level. Aqueous formic acid (0.2 ml/min, pH 3.5) was used as the mobile phase with a Develosil C30-UG column (Nomura Chemical Co., Ltd., Tokyo, Japan; 5 µm, 250 mm × 2.0 mm). Negative ionization was performed at −3.2 kV using an electrospray probe. For identification and quantification of each compound, multiple reaction monitoring (MRM) measurements were obtained. Optimized combinations of product and precursor ions for PA and OUA were determined as −42/−113 and −59/−131 respectively. Chromatographic retention times for PA and OUA were 6.0 and 10.5 min, respectively.

## Results and Discussion

### Identification of ^1^O_2_-induced oxidation products of UA

**Fig. 2:**
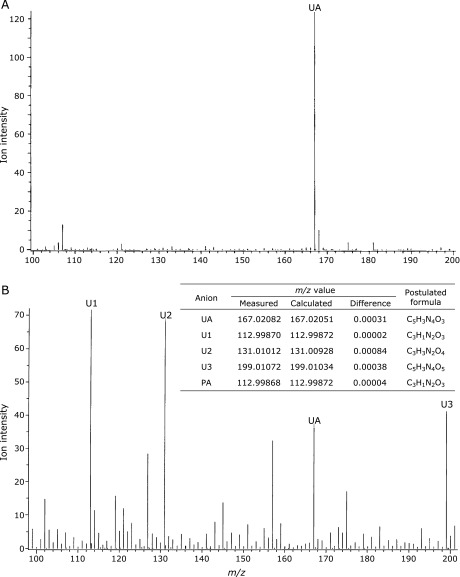
MS spectra of 200 µM UA (A) before and (B) after 60-min photooxidation induced by UVA irradiation (1.12 mW/cm^2^) using 10 µM Rose Bengal as a sensitizer. The TOFMS analysis was conducted in negative ESI mode with an ionization potential of −2,000 V. The measured *m/z* values were corrected using TFA as an internal standard. The chemical formula of PA and its candidates of U1, U2 and U3 are shown in the insert table with measured and calculated *m/z* values.

^1^O_2_ was produced from UVA irradiated 10 µM Rose Bengal. Figure 2 shows the changes in the MS spectra in the presence of 200 µM UA before (Fig. 2A) and 60 min after irradiation (Fig. 2B), as determined by negative electrospray ionization (ESI) mode TOFMS. The UA concentration was reduced to 35% and three unidentified anions, U1, U2 and U3, appeared in the MS spectrum after 60 min. These products were not seen in the absence of UA, suggesting they were derived from UA. The *m/z* value of U1 was determined to be −112.99870 using TFA as an internal standard, and its chemical formula was postulated to be C_3_HN_2_O_3_. This chemical formula is identical with that of PA and the *m/z* value of authentic PA is −112.99868. Furthermore, the retention times of U1 and authentic PA were identical (data not shown). We therefore concluded that U1 is PA.

The chemical formula of U2 was also determined to be C_3_H_4_N_2_O_4_, by its *m/z* value. This chemical formula is identical with OUA, a hydrolysate of PA. The retention times and MS spectra of U2 and authentic OUA were identical (data not shown), indicating that U2 is OUA. U3 (C_5_H_4_N_4_O_5_) was shown to be an O_2_ adduct of UA.

### Hydrolysis of PA to OUA

**Fig. 3:**
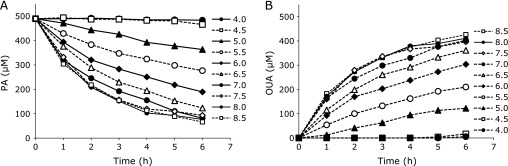
Hydrolysis of (A) PA to (B) OUA at room temperature at various pHs (4.0–8.5).

The effect of pH on the stability of aqueous PA was examined. The rates of PA hydrolysis and OUA formation increased with increasing pH (Fig. 3). The formation of OUA was stoichiometric with the decomposition of PA. The OUA formed was stable in solution at all pHs (4–8.5) for at least 1 week (data not shown).

The above results indicate that the ^1^O_2_-induced oxidation products of UA are PA and its hydrolysate OUA. We next examined whether this is true in other ^1^O_2_ formation systems such as thermal decomposition of NEPO and H_2_O_2_ plus ClO^−^ or ONOO^−^.

### Time course changes in PA and OUA levels during the oxidation of UA in various ^1^O_2_ production systems

**Fig. 4:**
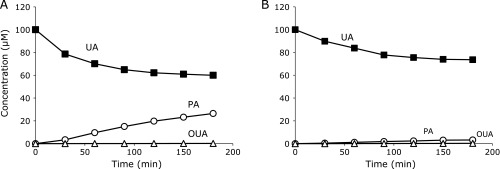
Oxidation of 100 µM UA (■) in methanol/H_2_O = 50/50 by ^1^O_2_ derived from 8.0 mM NEPO at 35°C and the formation of PA (◯) and OUA (△) in the absence (A) and in the presence (B) of 1.0 mM NaN_3_. All data are expressed as mean ± SD (*n* = 3).

**Fig. 5:**
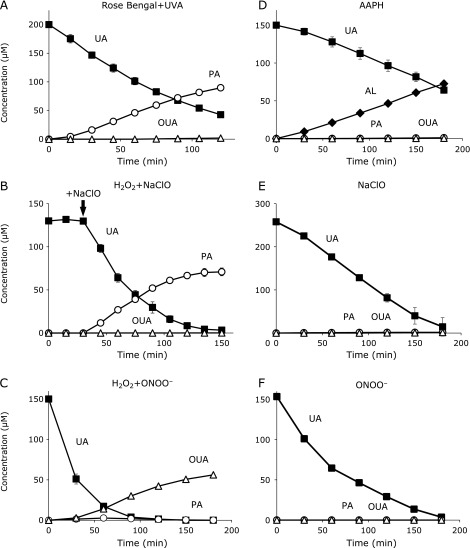
Oxidation of UA (■) and the formation of PA (◯), OUA (△), and AL (◆). All data are expressed as mean ± SD (*n* = 3). (A) UVA-induced photooxidation of 200 µM aqueous UA in the presence of 10 µM Rose Bengal. (B) Oxidation of 130 µM UA by ^1^O_2_ produced from 2.5 mM H_2_O_2_ and 300 µM NaClO in 100 mM phosphate buffer (pH 7.4) at room temperature. Constant addition of NaClO (2 µM/min) was started at 30 min. (C) Oxidation of 150 µM UA by ^1^O_2_ produced from 2.5 mM H_2_O_2_ and 1.0 mM SIN-1 (ONOO^−^ generator) in 100 mM phosphate buffer (pH 7.4) at room temperature. (D) Oxidation of 150 µM aqueous UA with peroxyl radicals produced from 10 mM AAPH at 37°C. (E) Oxidation of 260 µM UA with 360 µM NaClO in 100 mM phosphate buffer (pH 7.4) at room temperature. The addition of NaClO was kept constant (2 µM/min). (F) Oxidation of 190 µM UA by 1.0 mM SIN-1 in 100 mM phosphate buffer (pH 7.4) at room temperature.

First, we employed NEPO which gives ^1^O_2_ by its thermal decomposition. Figure 4A shows time course changes in UA, PA and OUA when 100 µM UA and 8 mM NEPO were incubated in methanol/water (50/50) at 35°C for 3 h. The major product was PA with a little OUA. The total yield of PA and OUA to consumed UA was 66.6%. When 1 mM NaN_3_, an ^1^O_2_ scavenger, was added to the reaction system, the rates of UA consumption and PA formation were reduced (Fig. 4B). The total yield of PA and OUA was also reduced to 13.2%, indicating that ^1^O_2_ is a key oxidant of UA. All the NEPO was decomposed within 4 h at 35°C. However we incubated the reaction solution for another 9 h and found an increase of PA and OUA formation (Table 1), indicating that intermediates such as U3 slowly decomposed to PA. Therefore, the total yield of PA and OUA increased to 99.1%, indicating that PA and OUA are the exclusive products of ^1^O_2_-induced oxidation of UA. This was also the case in the UVA-irradiated Rose Bengal system (Fig. 5A and Table 1).

Since all NEPO was converted to ^1^O_2_, we knew how much ^1^O_2_ was produced. We could then calculate the ratio of the amount of UA consumed to the total amount of ^1^O_2_ generated in the system. This ratio was 1/370 or 1/180, respectively, when 50 or 100 µM UA was oxidized (Table 1), indicating that ^1^O_2_ was predominantly quenched physically by UA or solvents. In fact, the rate constant for the quenching of ^1^O_2_ by UA was reported to be 3.6 × 10^8^ M^−1^·s^−1^,^(23)^ while the rate constant for the reaction of ^1^O_2_ with UA was determined to be 2.3 × 10^6^ M^−1^·s^−1^.^(24)^

The mechanism of PA formation remains unclear. The isolated U3 decomposed under neutral conditions to form PA (data not shown), suggesting that U3 is a direct precursor of PA formation. Identification of U3 is under investigation.

Hydrogen peroxide is known to be converted to ^1^O_2_ by the reaction with ClO^−^.^(20,21)^ To confirm this, we incubated aqueous UA in the presence of H_2_O_2_ with ClO^−^. Figure 5B shows the time course changes in UA, PA and OUA when 130 µM UA was incubated with 2.5 mM H_2_O_2_ and NaClO. NaClO was added at a rate of 2 µM/min from 30 min. Thereafter, UA was decreased and PA was increased. The total yield of PA and OUA reached 56.1% for 2.5 h oxidation. In the absence of H_2_O_2_, the rate of UA consumption was slower and a little formation of PA and OUA (1.3% yield) was observed (Table 1 and Fig. 5E). These results suggest that ClO^−^-induced oxidation of UA only produced slight PA and OUA and ClO^−^ converted H_2_O_2_ to ^1^O_2_.

Similarly, ONOO^−^ converts H_2_O_2_ to ^1^O_2_.^(25,26)^ Figure 5C shows the time course changes in UA, PA and OUA when 150 µM UA was incubated with 2.5 mM H_2_O_2_ and 1.0 mM SIN-1, an ONOO^−^ generator. The major product was OUA rather than PA because the pH of the reaction solution was ~8 which accelerated the hydrolysis of PA. The total yield of PA and OUA reached 37.0% for 3 h oxidation. In the absence of H_2_O_2_, the rate of UA consumption became slower and no formation of PA and OUA (0% yield) was observed (Table 1 and Fig. 5F). These results suggest that ONOO^−^-induced oxidation of UA produced no PA and OUA and ONOO^−^ converted H_2_O_2_ to ^1^O_2_. It is noteworthy that similar results were obtained when synthetic ONOO^−^ was used instead of SIN-1 (data not shown).

### Oxidation of UA induced by peroxyl radical, ClO^−^, or ONOO^−^

Thermal decomposition of AAPH produces two *tert*-carbon-centered radicals which are immediately converted to two *tert*-peroxyl radicals. Peroxyl radical-induced oxidation of aqueous UA resulted in UA decay and AL formation (Fig. 5D). The total yield of PA and OUA was only 1.9% but this was significant. This may suggest that a small amount of ^1^O_2_ was formed by the termination of two *tert*-peroxyl radicals,^(27)^ and/or the Russell-reaction of two methylperoxyl radicals formed by β-scission of *tert*-alkoxyl radical occurred.^(27–29)^ However, this requires further investigation.

As shown before, the total yield of PA and OUA in ClO^−^ and ONOO^−^-induced oxidation of UA was below 2%. Therefore, we concluded that PA is the ^1^O_2_ specific oxidation product of uric acid. We next tried to detect PA in biological samples.

### Detection of PA on human skin surface

Human skin surface was selected as a candidate of PA detection since UA is present there and the level of squalene hydroperoxide (^1^O_2_ oxidation product of squalene) increases after sunlight exposure.^(30)^ Methanol extracts of human skin were analyzed by LC/MS/MS. The analysis revealed the presence of UA and PA in skin lavage samples, but no OUA was detected. It is interesting that the PA and UA levels increased upon sunlight exposure (Table 2). The latter should be a protective response of human skin surface against photooxidation.

We are currently applying this method to human plasma samples. We believe our method is useful to determine the importance of ^1^O_2 _and its significance in many diseases under oxidative stress.

## Conclusions

We identified PA as the ^1^O_2_ specific oxidation product of UA. PA is slowly hydrolyzed to OUA under neutral conditions. Therefore, PA and OUA can serve as novel ^1^O_2_ markers *in vivo*. We detected PA on human skin surface and its level increased upon sunlight exposure, indicating that sunlight exposure induced the formation of ^1^O_2_ on human skin surface.

## Figures and Tables

**Table 1:** Formation of PA and OUA and their yields during UA oxidation induced by different types of ROS [µM, mean ± SD (*n* = 3)]

ROS	[UA]_0_	Time (h)	−Δ [UA]	[PA]	[OUA]	[PA] + [OUA]	Yield (%)
^1^O_2_ from 8.0 mM NEPO	50	12	21.3 ± 1.3	20.4 ± 1.3	0.29 ± 0.07	20.7 ± 1.4	97.0 ± 2.0
^1^O_2_ from 8.0 mM NEPO	100	3	40.0 ± 2.2	26.4 ± 0.9	0.18 ± 0.01	26.6 ± 0.9	66.6 ± 1.3
^1^O_2_ from 8.0 mM NEPO + 1.0 mM NaN_3_	100	3	26.4 ± 0.4	3.21 ± 0.04	0.25 ± 0.01	3.5 ± 0.04	13.2 ± 0.2
^1^O_2_ from 8.0 mM NEPO	100	12	44.6 ± 1.1	42.4 ± 0.8	0.57 ± 0.13	44.2 ± 1.2	99.1 ± 0.3
^1^O_2_ from UVA-irradiated Rose Bengal	50	12	50	7.2 ± 0.2	41.6 ± 0.4	48.8 ± 0.4	97.6 ± 0.8
^1^O_2_ from UVA-irradiated Rose Bengal	100	12	100	18.3 ± 0.4	78.6 ± 0.5	96.9 ± 0.2	96.9 ± 0.2
^1^O_2_ from UVA-irradiated Rose Bengal	150	12	150	33.1 ± 0.2	109.4 ± 0.1	142.5 ± 0.3	95.0 ± 0.2
^1^O_2_ from UVA-irradiated Rose Bengal	200	2	157 ± 3.4	89.7 ± 3.6	1.9 ± 1.4	91.7 ± 5.0	58.4 ± 4.3
^1^O_2_ from UVA-irradiated Rose Bengal	200	12	200	55.7 ± 0.7	129.8 ± 0.1	185.4 ± 0.7	92.7 ± 0.4
^1^O_2_ from 2.5 mM H_2_O_2_ + 300 µM ClO^−^	130	2.5	127 ± 0.5	70.9 ± 3.8	0.12 ± 0.003	71.0 ± 3.8	56.1 ± 3.3
^1^O_2_ from 2.5 mM H_2_O_2_ + ONOO^−^ (1.0 mM SIN-1)	150	3	150	0.04 ± 0.01	56.2 ± 0.3	56.3 ± 0.3	37.0 ± 0.2
Peroxyl radicals from 10 mM AAPH	150	3	85.9 ± 4.2	1.2 ± 0.1	0.5 ± 0.1	1.7 ± 0.1	1.9 ± 0.1
ClO^−^ (360 µM)	260	3	243 ± 9.9	1.6 ± 0.2	1.5 ± 0.2	3.1 ± 0.2	1.3 ± 0.1
ONOO^−^ (1.0 mM SIN-1)	200	3	132 ± 15	ND	ND	ND	0

ROS, reactive oxygen species; UA, uric acid; PA, parabanic acid; OUA, oxaluric acid; NEPO, 3-(1,4-dihydro-1,4-epidioxy-4-methyl-1-naphthyl)propionic acid; SIN-1, 3-(4-morpholinyl)sydnonimine, hydrochloride; ND, not detected.

**Table 2:** PA formation and UA secretion on human forearm skin surface exposed to sunlight for 2 h

PA (pmol/cm^2^)			UA (pmol/cm^2^)		
Before exposure	After exposure	After/Before	Before exposure	After exposure	After/Before
0.020 ± 0.010	0.065 ± 0.040*	3.1 ± 1.2	13.9 ± 15.1	46.2 ± 30.8	4.8 ± 4.2

PA, parabanic acid; UA, uric acid; Each value represents mean ± SD (*n* = 5); *p<0.05.

## Abbreviations

AAPH: 2',2-azobis(2-amidinopropane) dihydrochloride
AL: allantoin
DTPA: diethylenetriamine-*N*,*N*,*N'*,*N''*,*N''*-pentaacetic acid
ESI: electrospray ionization
NEPO: 3-(1,4-dihydro-1,4-epidioxy-4-methyl-1-naphthyl)propionic acid
OUA: oxaluric acid
PA: parabanic acid
ROS: reactive oxygen species
SIN-1: 3-(4-morpholinyl)sydnonimine, hydrochloride
TFA: trifluoroacetic acid
TOFMS: time-of-flight mass spectrometry
UA: uric acid
